# Interplay between cognitive conditions and individual traits in voluntary apnea

**DOI:** 10.1007/s00221-026-07244-7

**Published:** 2026-02-17

**Authors:** Eleonora Malloggi, Enrica L. Santarcangelo, Ursula Debarnot

**Affiliations:** 1 Department of Physics, University of Trento, Trento, Italy; 2 Department of Translational Research and New Technologies in Medicine and Surgery, University of Pisa, Via San Zeno 31, 56127 Pisa, Italy; 3 Laboratoire de Biologie de La Motricité, Université Claude Bernard Lyon 1, Lyon Villeurbanne, France

**Keywords:** Breath-holding, Motor imagery, Interoceptive sensibility, Hypnotizability, Absorption

## Abstract

Breath-holding is employed to enhance performance in sports such as diving, due to its specific physiological correlates. Its duration can be enhanced through training and specific cognitive strategies, and can be influenced by individual traits. Since hypnotizability has been linked to interoceptive sensibility and imagery abilities—both influencing breath-holding—we aimed to assess its contribution to breath-holding duration. The study investigates breath-hold duration during imagery of normal breathing (motor imagery, a cognitively incongruent condition) and while focusing attention on apnea sensations (internally focused attention, a cognitively congruent condition) in participants with different hypnotizability scores. Hypnotizability was assessed in healthy participants—as low-to-medium (med-lows, N = 16) and medium-to-high hypnotizables (med-highs, N = 17)—according to the Stanford Hypnotic Susceptibility Scale: Form A. They completed the Multidimensional Assessment of Interoceptive Awareness and the Movement Imagery Questionnaire-3 and performed three breath-holding trials of each condition in counterbalanced order. Heart rate and skin conductance were monitored. The vividness and ease of motor imagery and internally focused attention tasks were reported. Breath-holding duration increased across trials independently from cognitive condition and hypnotizability. Heart rate increased during motor imagery more than during internally focused attention, skin conductance decreased quasi-significantly during both tasks in both groups. Imagery abilities and interoceptive sensibility masked hypnotizability-related differences in the tasks’ subjective experience, heart rate, and increase in breath-holding duration across trials. Thus, hypnotizability, imagery abilities, and interoceptive sensibility jointly contribute to increase breath-holding duration across trials and to the associated experience and autonomic responses.

## Introduction

Voluntary apnea, also known as breath-holding, represents a specific physiological state characterized by coordinated autonomic responses, including bradycardia, increased mean arterial blood pressure, peripheral vasoconstriction (Ferrigno and Lundgren 2003; Sheel and Foster 2005), and reduced hemoglobin saturation (Ichinose et al. 2018). These changes reflect the diving reflex, an oxygen-conserving response that aims to maintain cerebral and cardiac perfusion under hypoxic stress. Vasodilator metabolites, such as adenosine, likely amplify this response; indeed, adenosine accumulates during hypoxia and induces bradycardia and vasodilation via A_1_/A_2_ receptors (Costalat et al. 2013; Marlinge et al. 2021). Breath-hold endurance is determined primarily by CO_2_ accumulation (Godfrey et al. 1969; Godfrey and Campbell 1969; Kelman and Wann 1971) and falling O_2_ , and is significantly affected by initial lung oxygenation (Bruce et al. 2021). Bruce and colleagues showed that pre-oxygenation prolongs apnea duration more than individual chemoreflex sensitivity (Bruce et al. 2021; Brown et al. 2024). The reported urge to breathe is positively correlated with the magnitude of the thoracic and abdominal expansion (Decavèle et al. 2025). Cognitive factors also modulate the urge to breathe, including time perception and mental tasks such as imagery of normal breathing and deep absorption in the breath-holding-related sensation (Vigran et al. 2019; Kanthack et al. 2019).

Although physiological limits ultimately govern apnea, the cognitive context can substantially influence performance. Attention and imagery tasks alter how long a person can hold their breath. When individuals engage in motor imagery of normal breathing during apnea, breath-hold duration increases compared to internally focusing on apnea sensations (Kanthack et al. 2019). This effect likely reflects a reduction in perceived threat, as imagining breathing may mitigate the discomfort normally associated with apnea (Kanthack et al. 2019). Conversely, directing attention toward breathlessness without imagery (internally focused attention, IFA) may intensify discomfort and reduce apnea tolerance. Thus, psychological factors such as attention, mental imagery, and temporal perception meaningfully affect a primarily physiological process (Vigran et al. 2019; Kanthack et al. 2019).

Breath-holding performance can be trained, as observed in swimmers and divers who can hold their breath for over 10 min and exhibit marked increases in cerebral blood flow (Arce-Álvarez et al. 2021; Bain et al. 2018).

### Motor imagery, hypnotizability and interoception

Motor imagery refers to the mental simulation of a movement without overt motor output (Decety 1996) and is used in sports to enhance performance (McNeil et al. 2025). It can be experienced either through internal visual imagery from a first-person perspective or through kinesthetic imagery, which involves vivid sensations of movement (Munzert et al. 2009). Motor imagery is characterized by subjective, behavioral and physiological variables that are influenced by imagery abilities (Guillot et al. 2009), motor conditions (Di Rienzo et al. 2022) and interoceptive sensibility (Malloggi et al. 2024). The latter represents the experience and interpretation of internal bodily signals (Critchley and Garfinkel 2017) and is measured by questionnaires (Mehling et al. 2018; Porges 1993). Motor imagery is also influenced by hypnotizability, a psychophysiological trait measured by scales that evaluate the ability to experience cognitive and physical states different from the actual ones (Santarcangelo and Zelic 2025). High hypnotizability is associated with greater functional equivalence between actual and imagined perception/action, that is more similar cortical activations and connections during the two conditions (Ibanez-Marcelo et al. 2019), and the greater excitability of their motor cortex during imagery (Spina et al. 2020; Cesari et al. 2020). Also, hypnotizability levels display different “adaptive” interoceptive sensibility (Diolaiuti et al. 2020) and interoceptive accuracy (Giusti et al. 2024), that is the ability to accurately detect interoceptive information, likely due to hypnotizability-related variations in the insula grey matter volume (Landry et al. 2017; Picerni et al. 2019).

### Aim of the study

In the general population, breath-holding duration is influenced by the concomitant cognitive context, with longer durations observed during motor imagery of normal breathing compared to internally focused attention (Kanthack et al. 2019).

Since imagery abilities, interoceptive sensibility, and hypnotizability are interrelated (Zelič et al. 2023), the present study aimed to expand the findings of Kanthack et al. (2019) by assessing whether these traits modulate breath-holding duration during two cognitive conditions: motor imagery of normal breathing (a cognitively incongruent task) and internally focused attention on apnea sensations (a cognitively congruent task). We hypothesize that individuals with higher imagery abilities and greater hypnotizability will benefit more from motor imagery, exhibiting longer breath-holding duration. Similarly, higher interoceptive sensibility may support greater tolerance during cognitively modulated breath-holding. In contrast, internally focused attention on breathlessness is expected not to enhance, and possibly to reduce breath-holding duration. By testing these hypotheses, the study aims to clarify how cognitive strategies and individual traits jointly shape physiological and autonomic responses during voluntary apnea.

## Methods

### Participants

Thirty-three healthy, sedentary volunteers (age mean ± SD): 27.2 ± 8.5; 15 females) were recruited among students at the University of Pisa. After signing an informed consent approved by the Pisa University Bioethics Committee (n.29/2022), a semi-structured interview ascertained the absence of cardiovascular, respiratory, neurological, psychiatric conditions, sleep and attention disorders, and any current pharmacological therapies. Participants were administered with the Italian version of the Hypnotic Susceptibility Scale: Form A (SHSS: A, range 0–12) (Weitzenhoffer and Hilgard 1959) and classified as highs (score ≥ 8 out of 12), mediums (score 5–7) and lows (score ≤ 4). In the general population, mediums represent 70%, while highs and lows represent 15% each (De Pascalis et al. 2000). Owing to the small number of recruited mediums, a group of medium-to-highs (med-highs including 2 mediums, 8 females, SHSS score (mean ± SD): 8.88 ± 1.32) and a group of medium-to-lows (med-lows, including 3 mediums, 7 females, SHSS score: 2.88 ± 1.82, 3 mediums) were studied.

For a preliminary assessment of the participants’ psychophysiological traits, participants completed the Multidimensional Assessment of Interoceptive Awareness (MAIA; Mehling et al. 2018) and the Motor Imagery Questionnaire-3 (MIQ-3; Williams et al. 2012). The MAIA questionnaire includes 8 scales (*noticing, not distracting, not worrying, attention regulation, emotional awareness, self-regulation, body listening, trusting*; range 0–5), while the MIQ includes three modalities (external visual, MIQEVI; internal visual, MIQIVI; kinesthetic imagery, MIQKI; total range 0–28). To study the effect of Trial and Task on experiential and behavioral variables (easiness and vividness, and apnea duration), which were studied during tasks, G*power (Faul et al. 2007) indicated a minimum number of 20 subjects to obtain significant main effects and interactions. To study the effect of Trial, Task and Level on physiological variables (SCL, HR and HRV), a minimum number of 14 subjects was needed. These effects were studied with repeated measures ANOVA within-between interaction model, and with f = 0.25, *p* = .05, and 1 − β = .80.

### Experimental procedure

The experiment was performed at least 1 week after the hypnotic assessment. The participants were asked to refrain from caffeine and alcohol for the 3 hours preceding the session, which was conducted in a sound and light-attenuated room. Before starting the experimental procedure, participants completed the State Anxiety Questionnaire (STAI-Y1; cut off for clinical anxiety = 52; Spielberger 1983). Before undergoing the experimental conditions, participants performed a familiarization trial of breath-holding until their breaking point, without cognitive instructions.

The experimental closed-eye session included 2-min baseline intervals (spontaneous, non-controlled breaths), following every trial of internally focused attention (B1) and motor imagery (B2) to let physiological signals return to baseline. Three maximal breath-hold trials were performed for internally focused attention and motor imagery in a seated position, with knees at 90° and with hands and forearms placed on the thighs. Before the breath-hold tasks (breath-holding), participants were instructed to take a deep breath. The inspiratory act was considered acceptable if its depth was at least one and a half times the normal breath, as controlled online by a Compumedics Summit IP® Inductive Respiratory Effort System (Compumedics, Newton, PA, USA). The duration of the breath hold was measured with a chronometer and controlled online by visualizing the respiratory signal. Participants were blinded to their maximal breath-hold duration.

In the internally focused attention condition, participants were instructed to focus on the bodily sensations that emerge during breath-holding. The experimenter provided guidance using instructions such as: “*Focus on the sensations associated with the effort of voluntary breath-holding. Feel the muscle contractions pressing against your lungs and the tension in your chest as it remains still. Notice the absence of movement in your respiratory tract and the growing urge to breathe*”*.* In the motor imagery condition, performed while imagining normal breathing, participants were instructed to engage in both visual and kinesthetic imagery of natural respiration. They were guided with instructions such as: “*Imagine the sensations associated with breathing. Feel the air entering your nose and the pressure changes in your lungs and chest. Sense the muscular contractions and stretches accompanying inhalation and exhalation, as if you were breathing*”. After each breath-holding trial, the participants were invited to rate the vividness and easiness of their internally focused attention and motor imagery experience.

Internally focused attention and motor imagery were randomly administered to the participants. Randomization was carried out via computer-generated random sequences, thus ensuring that both conditions (internally focused attention and motor imagery) were counterbalanced within each hypnotizability group.

After each breath-holding trial, the participants were invited to rate the vividness and easiness of their internally focused attention and motor imagery experience.

### Signal acquisition and analysis

Electrocardiogram (ECG) and skin conductance level (SCL) were monitored by a Psylab device (Contact Precision Instrument) and stored for offline analysis. For ECG acquisition, disposable electrodes (https://www.fiab.it/it/index.php) were placed according to the standard DI configuration. SCL was recorded using two similar electrodes placed on the middle phalanges of the index and middle fingers of the dominant hand. A common reference electrode was used for both ECG and SCL signals. Physiological signals were acquired at a sampling rate of 250 Hz. They underwent artifact inspection before analysis. ECG traces were visually inspected for noise, movement artifacts, and ectopic beats. Artifactual R-peaks were corrected using cubic-spline interpolation, and segments containing more than 5% artifacts were excluded from heart rate variability (HRV) analysis. Skin conductance abrupt variations exceeding 3 standard deviations from the average, computed over a 2-s sliding window, were classified as artifacts. A band-pass filter (0.5 Hz–20 Hz) was applied to the ECG signal, and heart rate (HR) was computed as 60/mean RR interval for each condition (B1, B2, internally focused attention, motor imagery). HRV was estimated using the root mean square of successive differences (RMSSD), a time-domain index reflecting short-term parasympathetic modulation. RMSSD values were interpreted in consideration of normative data for short-term resting conditions (Nunan et al. 2010). Notably, total HRV (SDNN) could not be studied due to the short duration of the studied conditions (Shaffer et al. 2017). For SCL, the signal was detrended, and its mean value was subtracted from the entire signal as baseline removal. A fourth-order low-pass butter filter was applied with a cut-off frequency set at 0.5 Hz. Then, a Savitzky-Golay smoothing filter was applied (Savitzky and Golay 1964). Breath-hold trials were averaged during internally focused attention and motor imagery, respectively, as well as baseline intervals. The mean value of HRV and SCL was computed for each condition.

### Variables

For the assessment of interoceptive sensibility and motor imagery abilities, we studied the MAIA and MIQ-3 scales. The variables studied during the experimental session were experiential (state anxiety, STAI-Y1, Spielberger 1983, vividness and easiness of internally focused attention and motor imagery, range 1 min–7 max), behavioral (breath-holding duration, s), and autonomic (HR, bpm, HR fast variability (RMSSD, ms), and SCL, μS). A reduced number of participants (N = 21, 10 med-highs and 11 med-lows) agreed to undergo the assessment of HR and SCL.

### Statistical analysis

The statistical Package SPSS 15 was used for all analyses. For the preliminary assessment, separate multivariate analyses of variance (MANOVAs) were conducted on the MAIA and MIQ-3 scales. For the experimental session, univariate ANOVA was applied to STAI-Y1 scores. Repeated measures ANOVAs were applied to breath-holding duration, vividness, and easiness of the cognitive task, according to a 2 groups (med-highs, med-lows) × 2 Tasks (internally focused attention, motor imagery) × 3 Trials (T1, T2, T3) design.

Repeated measures ANOVAs were applied to HR, RMSSD, and SCL following a 2 Group (med-highs, med-lows) × 3 Trial (T1, T2, T3) × 2 Task (internally focused attention, motor imagery) × 2 Level (baseline, task). The Greenhouse–Geisser ε correction was used for non-sphericity. Contrast analysis between levels was performed. ANCOVAs were applied to breath-holding duration, vividness, easiness, HR, RMSSD, and SCL, controlling MIQ-3 and MAIA dimensions.

In addition, linear backward regression analysis was performed to detail the magnitude of the effects of hypnotizability, MAIA and MIQ scores, HR and SCL on the breath-holding duration.

## Results

### Questionnaires

**Fig. 1:**
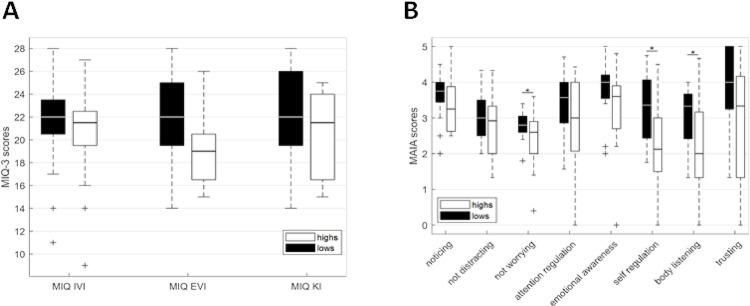
Imagery abilities and interoceptive scores (mean, SD). **A** MIQ; MIQIVI, internal visual imagery, MIQEVI, external visual imagery; MIQKI, Kinesthetic imagery. **B** MAIA scales. (b) Lines indicate significant differences between groups (*p < .05)

MIQ-3 scores (Cronbach’s alpha = 0.63) were not significantly different between groups (*F*(7,30) = 1.50, *p* = .23) (Fig. 1A). MAIA scales (Cronbach’s alpha = 0.89) did not exhibit a significant group effect (*F*(7,30) = 1.47, *p* = .22). Nonetheless, *not worrying* (*F*(1,30) = 4.42, *p* = .044), *self-regulation* (*F*(1,30) = 7.81, *p* = .009) and *body listening* (*F*(1,30) = 4.63, *p* = .040) differed between med-highs and med-lows (Fig. 1B). However, when applying Bonferroni correction for multiple comparisons (adjusted α = .006), none of the univariate effects remained significant.

STAI-Y1 scores did not differ between groups (mean ± SD; med-highs, 31.59 ± 8.96; med-lows, 33.18 ± 8.41) and were in the normality range (< 52).

### Breath-holding duration

**Fig. 2:**
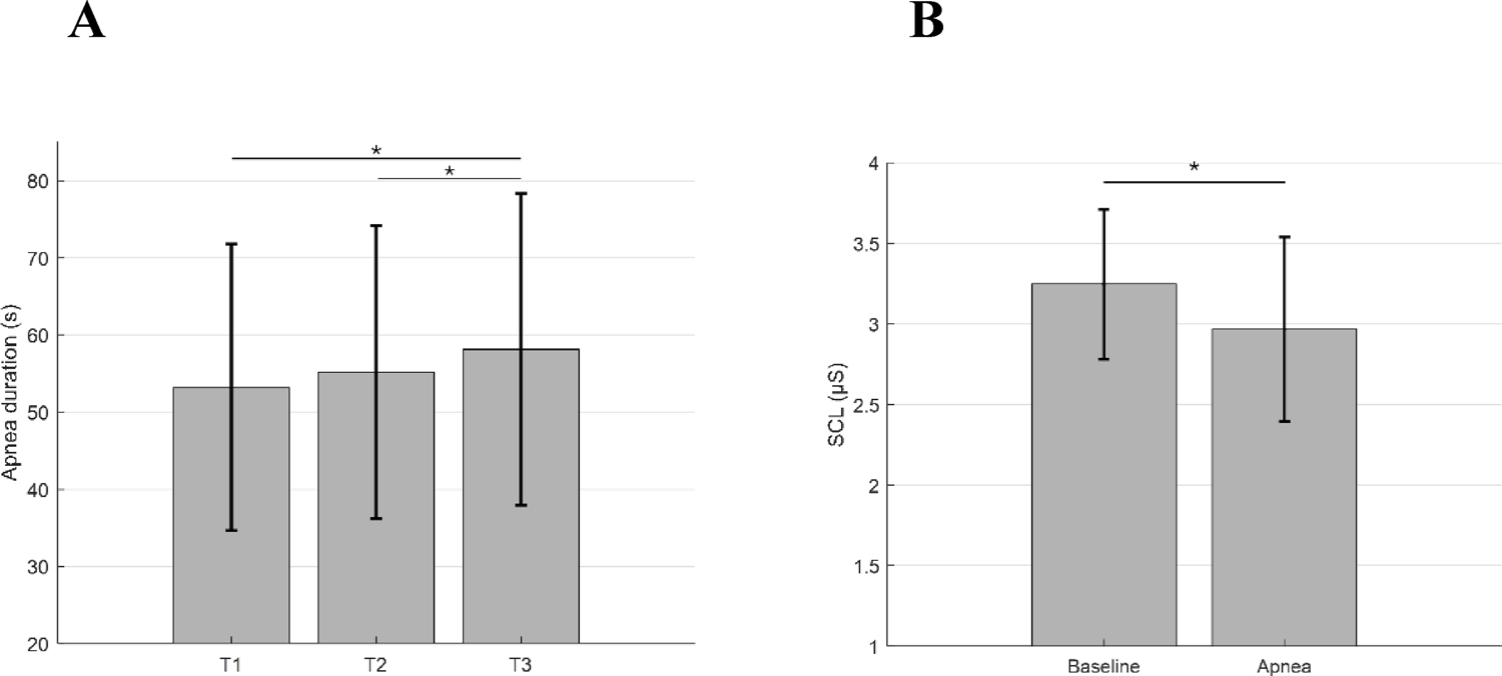
Breath-holding duration and SCL (mean, SD). **A** Breath-holding duration (s) in each trial independently from conditions; **B** SCL level (μS) during Baseline and breath-holding, independently from the type of trials. Lines indicate significant differences (*p < .05)

Repeated measures ANOVA revealed only a significant Trial effect (*F*(2,38) = 3.79, *p* = .032, η^2^ = .166, 1 − ß = .656) indicating increasing breath-holding duration across consecutive trials (T1 < T3, (*F*(1,19) = 8.21, *p* = .010; T2 < T3 (*F*(1,19) = 4.13, *p* = .056) independently from tasks and hypnotizability (Fig. 2A). Estimated marginal means increased from T1 (M = 53.24 s, 95% CI [46.55, 59.94]) to T2 (M = 55.21 s, 95% CI [48.37, 62.05]) and T3 (M = 58.17 s, 95% CI [50.89, 65.45]). Both imagery abilities and interoceptive sensibility accounted for these learning effects, as controlling for MAIA and MIQ-3 dimensions separately abolished the Trial effect.

### Self-reported easiness and vividness of internally focused attention and motor imagery

The reported easiness (internally focused attention (mean ± SD): 5.29 ± 1.09; motor imagery: 4.41 ± 1.47) of the tasks exhibited a significant Task effect (*F*(1,31) = 7.17, *p* = .012, η^2^ = .188, 1 − ß = .737), indicating greater easiness to perform internally focused attention (M = 5.17, 95% CI [4.76, 5.58]) than motor imagery (M = 4.41, 95% CI [3.90, 4.93]). Controlling ANOVA for MIQIVI disclosed a Trial effect (*F*(2,60) = 3.49, *p* = .037, η^2^ = .104, 1 − ß = .631), with T2 < T3 (*F*(1,30) = 7.85, *p* = .009). Controlling it for MIQKI disclosed a Task × Group interaction (*F*(1,30) = 4.14, *p* = .051, η^2^ = .121, 1 − ß = .504). Its decomposition revealed that med-highs report greater easiness than med-lows for motor imagery (*F*(1,31) = 6.56, *p* = .015). Controlling ANOVA for all MAIA dimensions, except for *not worrying,* abolished the Task effect.

The reported vividness (internally focused attention, mean ± SD: 5.20 ± 1.21; motor imagery: 4.45 ± 1.48) differed between Trials (*F*(2,62) = 5.10, *p* = .009, η^2^ = .141, 1 − ß = .804) and increased from T1 (M = 4.63, 95% CI [4.24, 5.01]) to T3 (M = 4.98, 95% CI [4.59, 5.37]) (T1 = T2, T2 < T3 (*F*(1,31) = 11.04, *p* = .002; T1 < T3 (*F*(1,31) = 5.70, *p* = .023), and indicated greater vividness for internally focused attention (M = 5.24, 95% CI [4.81, 5.67]) than motor imagery (M = 4.25, 95% CI [3.69, 4.81]) (Task effect, *F*(1,31) = 8.67, *p* = .006, η^2^ = .219, 1 − ß = .813).

Controlling for MAIA noticing, not distracting, attention regulation, trusting, MIQEVI, and MIQIVI abolished both effects. Controlling for *not worrying, emotional awareness,* and *self-regulation* abolished only the Trial effect. Controlling for MIQKI abolished both effects and disclosed a Task × Group interaction (*F*(1,30) = 4.88, *p* = .035, η^2^ = .140, 1 − ß = .571). Its decomposition revealed a difference between tasks only in med-lows, who exhibited higher vividness for internally focused attention than motor imagery (*F*(1,15) = 8.21, *p* = .012).

### Physiological variables

HR did not exhibit any significant differences between groups (*F*(1,19) = 1.32, *p* = .26), trials (*F*(2,38) = 0.27, *p* = .76) and tasks (*F*(1,19) = .30, *p* = .59) (mean ± SD; baseline: 72.74 ± 11.21; task: 71.88 ± 11.41). Controlling for MAIA *not distracting* disclosed a Task × Level interaction (*F*(1,18) = 7.58, *p* = .013, η^2^ = .296, 1 − ß = .739), whose decomposition revealed lower HR during internally focused attention than motor imagery (*F*(1,19) = 13.29, *p* = .002) in the presence of a non-different baseline.

RMSSD (ms) did not exhibit significant effects and interactions (mean ± SD; B: 92.78 ± 70.13; task: 146.72 ± 162.98).

Mean SCL exhibited a significant Trial effect (*F*(2,38) = 3.16, *p* = .054, η^2^ = .143, 1 − ß = .571), with T1 = T2, T1 (M = 4.20, 95% CI [4.02, 4.37]) < T3 (M = 4.08, 95% CI [3.95, 4.22]) (*F*(1,19) = 4.52, *p* = .047), and T2 = T3. The Level effect (*F*(1,19) = 6.11, *p* = .023, η^2^ = .243, 1 − ß = .650) consisted only of higher SCL in B than in tasks independently of the cognitive condition (Fig. 2B). Controlling for all MAIA dimensions abolished all effects. Controlling for MIQIVI and MIQEVI abolished all effects. MIQKI abolished all effects and disclosed a Trial × Task × Level × Group interaction (*F*(2,36) = 3.90, *p* = .029, η^2^ = .178, 1 − ß = .667). Its decomposition revealed a Level effect in med-highs (*F*(1,8) = 7.12, *p* = .028), with B > Task. In med-lows it revealed a Level × Task interaction (*F*(1,9) = 12.03, *p* = .007), with B > internally focused attention in T1 (*F*(1,9) = 15.60, *p* = .003), and no significant effects in T2 and T3.

### Regression analysis of SHSS and MAIA scores, and physiological variables on breath-holding duration

After controlling collinearities, linear backward regression of MAIA and MIQ dimensions, HR, and SCL on breath-holding duration was performed, and is reported in Table 1. It indicates the role of a few MAIA dimensions in breath-holding duration in the first trial of both internally focused attention and motor imagery, and an increasing role of MAIA dimensions and autonomic variables in the successive trials during internally focused attention, but not during motor imagery.

**Table 1:** Linear backward regression

Breath-holding duration	Internally focused attention				Motor imagery			
	R^2^		B	*p*	R^2^		B	*p*
T1	0.198	Noticing	− 0.468	.045	0.241	Noticing	− 0.604	.014
		Trusting	0.484	.039		Attention regulation	0.486	.043
T2	0.526	SHSS	− 0.797	.003				
		Noticing	− 1.09	.0001				
		Emotional awareness	0.710	.009				
		Body listening	0.984	.001				
		SCL	− 0.886	.002				
T3	0.362	Noticing	− 0.587	.013				
		Attention regulation	0.743	.004				
		RMSSD	− 0.453	*.052*				
		HR	− 0.539	.026				

B, standardized beta; HR, heart rate; R^2^, adjusted R^2^; RMSSD, heart rate fast variability; SCL, skin conductance level; SHSS, hypnotizability score; T1, T2, T3, first, second, and third trial

## Discussion

The present study was designed to explore how hypnotizability, imagery abilities, and interoceptive sensibility influence breath-hold performance in different cognitive conditions. Our findings indicate that breath-holding duration did not differ significantly between the cognitively congruent and incongruent conditions. Additionally, attention to breath-holding sensations (congruent with the physical state) and motor imagery of normal breathing (incongruent with it) are differentially associated with experiential and physiological variables.

The findings reveal a dissociation between subjective experience and breath-holding duration, as interoceptive sensibility and autonomic markers are more strongly associated with breath-holding duration in the congruent cognitive condition than in the incongruent condition. Interoceptive sensibility and motor imagery abilities account for most of the differences between the two conditions, and mask hypnotizability-related differences.

### Subjective experience

The study did not demonstrate significant hypnotizability-related differences in the self-reported scores of trait imagery abilities (measured by the Betts’ questionnaire), in line with the observation that questionnaire scores do not always correlate with hypnotizability (Srzich et al. 2016) and with functional equivalence between actual and imagined perception/action (Ibanez-Marcelo et al. 2019).

Med-highs and med-lows reported different experiences in cognitively congruent and incongruent breath-holding conditions. The higher vividness of the congruent than the incongruent experience observed in both groups suggests that the former was easier than the latter. In contrast, the greater easiness of the cognitively incongruent task in med-highs was masked by the trait kinesthetic ability of imagery, which compensated for the med-lows’ lower imagery ability and weaker functional equivalence (Ibanez-Marcelo et al. 2019). Two non-alternative hypotheses can account for the groups’ different experiences. One can be based on the highs’ flexible functional connections between the salience and executive systems (Hoeft et al. 2012), the other can be sustained by the highs' lower ability to represent bodily signals accurately (Giusti et al. 2024).

Interoceptive sensibility contributed to the difference in task easiness. The ability to allocate attention (*attention regulation*) and adaptively modulate internal bodily sensations of discomfort (*self-regulation*), together with *emotional awareness,* likely induced a more vivid experience during the cognitively congruent breath-holding condition. Enhanced vividness also depends on the association between respiratory sensations and emotional processes (Harrison et al. 2021), making them more easily accessible or meaningful during imagery in emotionally resonant contexts such as breath-holding. 

### Breath-holding duration

Hypnotizability did not influence breath-holding duration in both cognitive conditions and in both groups, despite the highs’ lower interoceptive accuracy (Giusti et al. 2024), which was expected to positively influence breath-holding duration when attention was focused on the breath-holding -related sensations, and their stronger functional equivalence between actual and imagined normal breathing, which was expected to increase breath-holding duration during imagination of normal breathing. The absence of behavioral hypnotizability-related differences between groups contrasts with their subjective experiences, as earlier observed for other functions, such as postural control, which was quite different between highs and lows but was similarly reported (Santarcangelo et al. 2008).

In contrast to the study by Kanthack et al. (2019), we did not find a significant differences in breath-holding duration between the cognitively congruent (internally focused attention) and incongruent (motor imagery) conditions. A possible explanation for this discrepancy lies in the composition of the two samples. While Kanthack et al.’s participants were recruited from the general population through a sports science department and were all physically active individuals engaged in regular sporting practices, our sample consisted of non-athletes with no systematic physical training. It is plausible that athletes are more familiar with motor imagery, either through formal practice or embodied experience, which may enhance the effectiveness of motor imagery in extending breath-holding duration. This hypothesis aligns with the well-established role of motor imagery in sports performance and motor learning (Desai et al. 2025). In contrast, our sample's limited familiarity with motor imagery, and its composition comprising many med-lows, which differs from the general population (De Pascalis et al. 2000), may have reduced the efficacy of motor imagery (Ibanez-Marcelo et al. 2019).

In line with earlier reports (Kanthack et al. 2019), the duration of breath-holding increased across trials. In our study, both interoceptive sensibility and imagery abilities contributed to this effect, which can be attributed to the observed cooperation of interoceptive sensibility in the cortical representation of both actual and imagined movements (Malloggi et al. 2024). Interestingly, no correlation emerged between breath-holding duration and the subjective experience of the two tasks, suggesting that performance improvements may have been more strongly influenced by arousal or emotional regulation than by cognitive engagement (Maric et al. 2020; Harrison et al. 2021).

### Physiological variables

In the general population, heart rate decreases during 30–60 s of normoxic breath-holding (O’Croinin et al. 2024). In this condition, earlier findings do not reveal significant differences in heart rate between breath-holding with instructions and with attention focused on bodily sensations (Kanthack et al. 2019), which could be attributed to the low cognitive effort required by the congruent condition. In the more demanding, cognitively incongruent condition, in contrast to earlier reports (Kanthack et al. 2019), we observed an increase in heart rate compared to baseline, consistent with the increase in the sympathetic/parasympathetic ratio observed during demanding cognitive tasks (Chang & Huang 2012). The MAIA *not distracting* dimension, not measured in the earlier study (Kanthack et al. 2019), may have masked the difference. The parasympathetic, fast heart rate variability (RMSSD) did not differ between tasks and hypnotizability groups, likely due to the different contributions to its regulation by multiple components, such as breath-holding, cognitive effort, emotion, and arousal, which may balance each other. Indeed, breath-holding is associated with both sympathetic and parasympathetic activation (Bain et al. 2018).

Our results also showed a reduction in skin conductance during breath-holding compared to baseline. While both physical and cognitive load are typically associated with increased skin conductance due to elevated sympathetic nervous system activity (Critchley 2002; Boucsein 2012; Chang and Huang 2012), voluntary breath-holding constitutes a unique autonomic condition. It triggers the diving reflex, a physiological response characterized by bradycardia, peripheral vasoconstriction, and reduced blood flow to the extremities of the limbs (Ferrigno and Lundgren 2003), which potentially leads to reduced skin conductance despite an elevated central sympathetic drive (Heusser et al. 2009). It is noteworthy that interoceptive sensibility sustained the observed SCL differences, supporting the idea that it modulates autonomic activity during challenging bodily states such as breath-holding.

### Limitations and conclusions

Limitations of the study are the sample's scarce representativeness of the general population (De Pascalis et al. 2000) and the classification of lows-to-mediums and mediums-to-highs based on their total hypnotizability scores rather than on a more accurate categorization based on motor inhibition, hallucination, and dissociation (Terhune et al. 2011). Another limitation is not having acquired, but only monitored online, the respirogram. Its acquisition could have allowed for the correlation of the amplitudes of maximal inspiration with the experiential, behavioral, and autonomic results, as different thorax enlargements correspond to different interoceptive conditions. However, the sample was substantially composed of females of comparable age who did not practice sports; thus, large differences between groups were not expected. Furthermore, the addition of a neutral breath-holding condition without employing cognitive strategies could have represented a control condition. Finally, we are aware that, after hypnotic induction (Schmidt et al. 2024), anxiety might have been reduced, thus likely influencing interoception.

In conclusion, the results support the view that, in healthy participants, interoceptive sensibility and trait imagery abilities are relevant to breath-holding duration and that hypnotizability contributes to its subjective and physiological correlates. The complexity of the interaction between these variables in regulating the effects of cognitive conditions on the physiological state of breath-holding suggests that assessing interoceptive and imagery abilities could be beneficial for sports performers, who may benefit from training in voluntary breath-holding (Bouten et al. 2024).

## Data Availability

Data are available at https://osf.io/knxt8/overview.
